# Supplementary figures and images for: DMPNet: densely connected multi-scale pyramid networks for crowd counting
Source: PeerJ Comput Sci. 2022 Mar 18;8:e902. doi: 10.7717/peerj-cs.902 (PMC9044264; doi:10.7717/peerj-cs.902)

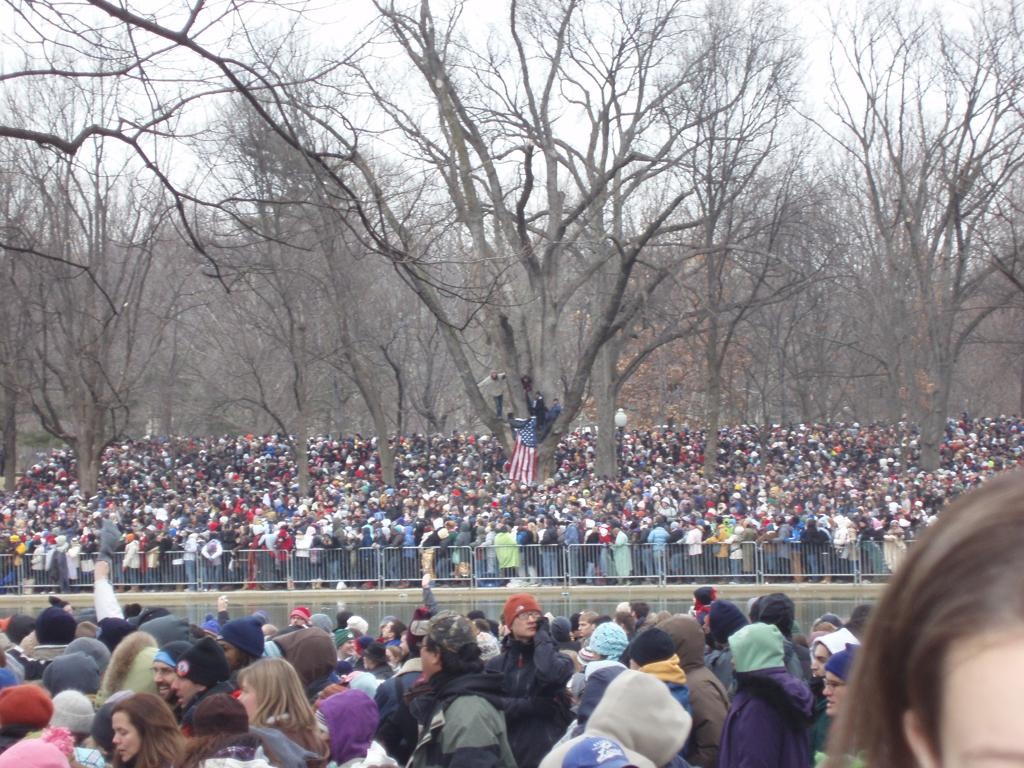

Supplement: Supplemental Information 1 [file peerj-cs-08-902-s001.tar › Code/IMG_1.jpg]

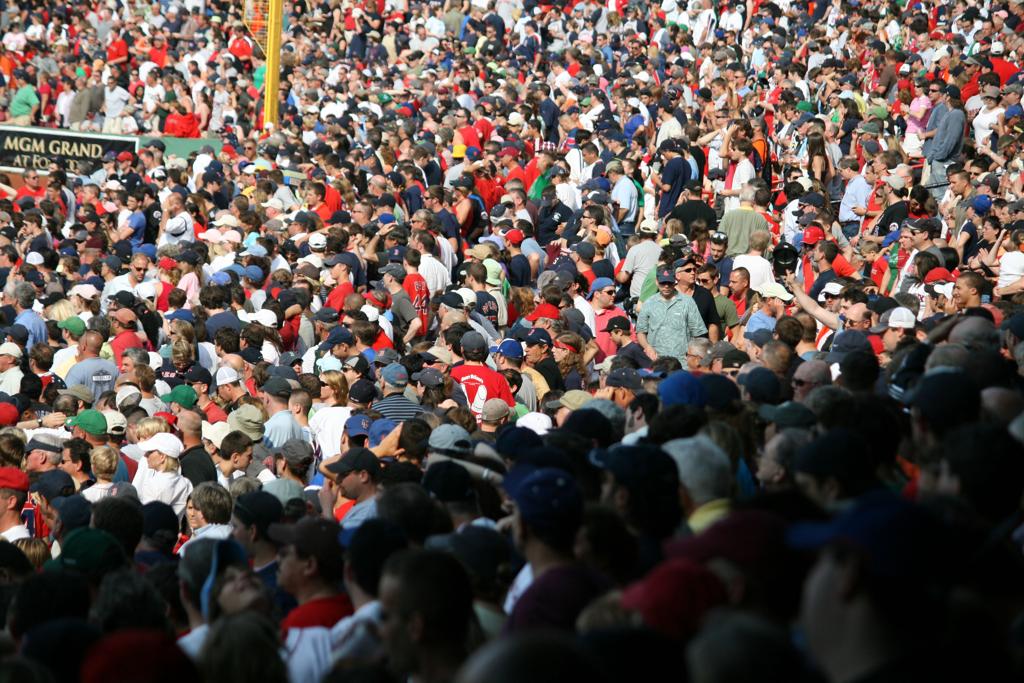

Supplement: Supplemental Information 1 [file peerj-cs-08-902-s001.tar › Code/IMG_10.jpg]
